# Sex Differences in Constitutive Autophagy

**DOI:** 10.1155/2014/652817

**Published:** 2014-02-27

**Authors:** Sara Oliván, Ana Cristina Calvo, Raquel Manzano, Pilar Zaragoza, Rosario Osta

**Affiliations:** LAGENBIO-I3A, Veterinary School, University of Zaragoza, Miguel Servet 177, 50013 Zaragoza, Spain

## Abstract

Sex bias has been described nowadays in biomedical research on animal models, although sexual dimorphism has been confirmed widely under pathological and physiological conditions. The main objective of our work was to study the sex differences in constitutive autophagy in spinal cord and skeletal muscle tissue from wild type mice. To examine the influence of sex on autophagy, mRNA and proteins were extracted from male and female mice tissues. The expressions of microtubule-associated protein 1 light chain 3 (LC3) and sequestosome 1 (p62), markers to monitor autophagy, were analyzed at 40, 60, 90, and 120 days of age. We found significant sex differences in the expression of LC3 and p62 in both tissues at these ages. The results indicated that sex and tissue specific differences exist in constitutive autophagy. These data underlined the need to include both sexes in the experimental groups to minimize any sex bias.

## 1. Introduction

In biomedical research, single-sex studies (generally males) still predominate. Moreover, a high number of studies either fail to specify the sex used or the two sexes are not analyzed separately. In neuroscience or pharmacology, for example, a higher number of studies are conducted in males (5.5 : 1 or 5 : 1) albeit gender differences have been previously described [1]. However, an increasing number of investigations about the influence of gender in human disorders (cancer, neurodegenerative, or cardiovascular diseases) or fundamental biological pathways, such as autophagy, are currently being carried out.

Autophagy is a dynamic system for degrading misfolded and/or damaged proteins and, therefore, for maintaining the cellular homeostasis [2]. During this process, small portions of cytoplasm are sequestered by a double membrane bound vesicles called autophagosomes and consequently degraded when they fuse with lysosomes to form an autolysosome. Cellular autophagic activity is usually low under normal physiological conditions (constitutive autophagy) but can be markedly upregulated by numerous stimuli (starvation, hypoxia, or infections) or suppressed as documented in the case of neurodegenerative disorders and cancer [3]. Specifically in neural cells, the role of constitutive autophagy has been studied and the results indicate that it is essential to prevent certain neurodegenerative diseases [4, 5].

Sex-dependent differences in the activation of the autophagic cytoprotection pathway have long been reported *in vitro*. During starvation, cultured male neurons readily undergo autophagy and die, whereas neurons from females mobilize fatty acids, accumulate triglycerides, form lipid droplets, and survive longer [6, 7]. However, only in few cases, the possible gender-associated differences in autophagy have been investigated *in vivo* and are still less in normal physiological conditions [7–9]. Recent studies have also shown not only a sexual dimorphism in autophagy but also tissue specificity in constitutive autophagy in adult rats [9].

The aim of this study was to investigate the sex differences in autophagy under normal physiological conditions in spinal cord and muscle tissue from wild type mice. RNA and protein level expression of the microtubule-associated protein 1 light chain 3 (LC3) and p62/sequestosome 1 (p62) were assessed, as these are the most commonly used markers to monitor autophagy. LC3 is associated with completed autophagosome, while p62 becomes incorporated into the completed autophagosome through its direct binding to LC3 and is subsequently degraded in the autolysosomes [9, 10].

## 2. Material and Methods

### 2.1. Mice

From each sex, twelve wild type mice (B6SJL) at postnatal (P) days 40, 60, 90, and 120 were used in the study. These selected ages corresponded to the progression of the sexual maturation of the animals and therefore this study could be carried out to follow accurately this progression. The Ethic Committee for Animal Experiments of the University of Zaragoza approved all experimental procedures. Animal care and experimentation were performed accordingly with the Spanish Policy for Animal Protection RD53/2013, which meets the European Union Directive 2010/63/UE on the protection of animals used for experimental and other scientific purposes. Food and water were available *ad libitum. *The mice were sacrificed by CO_2_ anaesthesia. Spinal cord and skeletal muscle were dissected, snap-frozen, and preserved at −80°C.

### 2.2. Real-Time-PCR

For RNA extraction, spinal cord and skeletal muscle tissue were pulverized in liquid nitrogen with a cell crusher using Trizol Reagent (Invitrogen). RNA was treated with Turbo DNA-free kit (Ambion) to eliminate genomic DNA. Reverse transcription was carried out according to the SuperScript First-Strand Synthesis System kit (Invitrogen). Gene expression was assayed by real-time PCR. Primer and probe mixture for LC-3 (Map1lc3a, Mm00458724_m1) and p62 (Sqstm1, Mm00448091_m1) were supplied by Applied Biosystems. Two endogenous genes (GAPDH: 4352932E and *β*-actin: 4352933E, Applied Biosystems) were used for normalization of the data [11]. Target gene expression was normalized using the geometric mean of these two housekeeping genes and relative gene expression was determined using the 2^−ΔΔCT^ method [12, 13]. All reactions were performed in triplicate and the primer/probe efficiencies of sets were close to 100%.

### 2.3. Western Blot

Powdered tissue was homogenized in RIPA lyses buffer with protease inhibitors (Complete, Roche); the homogenate was centrifuged at 10000 ×g for 10 min at 4°C and the resulting supernatants were collected. Protein concentration was determined by BCA method (Sigma-Aldrich). Forty micrograms of total protein was subjected to SDS/PAGE and transferred to PVDF membranes (Amersham Biosciences). Membranes were incubated in blocking solution overnight at 4°C and then incubated one hour with primary antibodies against LC-3 (1:1000, MBL), p62/SQSTM1 (1:1000, Enzo Life Sciences), and GAPDH (1:1000, Santa Cruz). After incubation with HRP-conjugated secondary antibodies (Santa Cruz), bands were visualized by enhanced chemiluminescent reagent (GE Healthcare Life Science). Immunoblots were exposed and scanned and densitometry was measured with AlphaEaseFC software (Bonsai).

### 2.4. Statistical Analysis

Different groups were compared using Student's *t*-test. This statistical analysis was carried out in SPSS version 19.0 software (IBM). The data are presented as means, and error bars represent the standard error of the mean (SEM). Significance was set at a *p* value of less than 0.05.

## 3. Results

### 3.1. RT-PCR in Muscle and Spinal Cord Tissue

**Figure 1:**
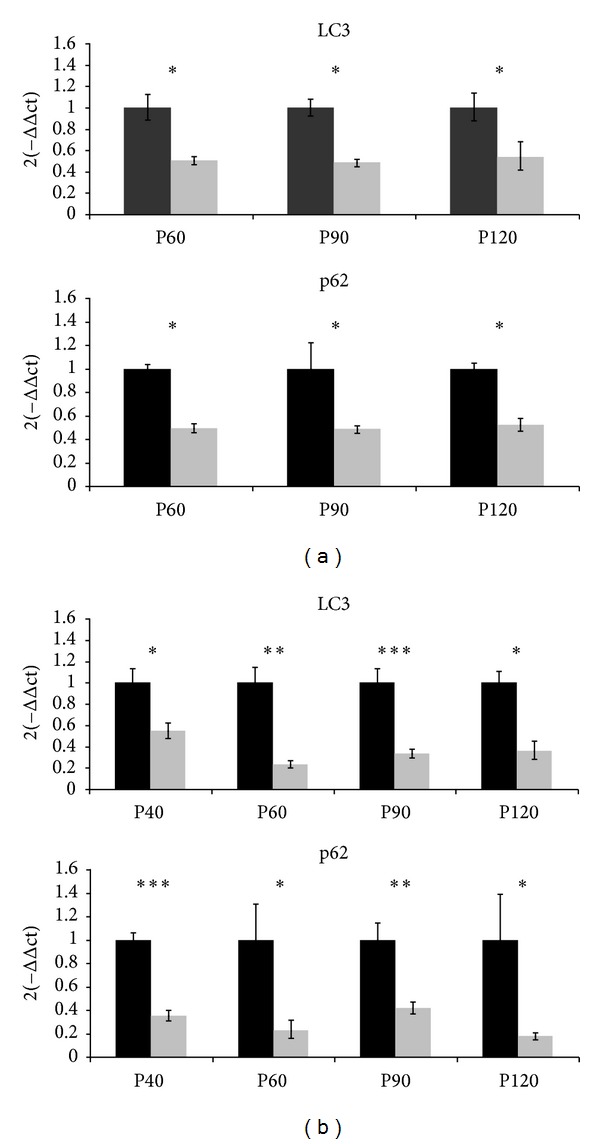
LC3 and p62 mRNA expression in the spinal cord and the skeletal muscle. (a) The transcript levels in the spinal cord of males (black bars) and females (grey bars) from mice at P60, P90, and P120 are shown. Relative expression values are females compared with males (set at 1) at each age. (b) The transcript levels in skeletal muscles of male (black bars) and female (grey bars) mice at P40, P60, P90, and P120 are shown as in (a). Each data point represents the mean ± SEM of twelve mice. Asterisks denote a student *t*-test *p* value <0.05 (*), <0.01 (**), and <0.001 (***).

At the transcriptional level in spinal cord tissue (Figure 1(a)), LC3 expression levels were generally lower in females than in males, and statistical differences were found at P60, P90, and P120 (*p < 0.05). p62 mRNA expression levels in females were lower than in males at all studied ages (*p < 0.05). Notably, the expression of both genes maintained constant proportional levels between the sexes at all different ages. In skeletal muscle tissue (Figure 1(b)), the mRNA expression pattern was similar to the one observed in spinal cord. In the case of LC-3 transcripts, females showed less LC-3 expression levels than males. The differences between the sexes were significant at each studied age, although the most significant difference was detected at P90 (***p < 0.001) and P60 (**p < 0.01). Also, the p62 gene expression was significantly decreased in females at all ages, and the most significant difference between sexes was at P40 (***p < 0.001) and P90 (**p < 0.01). 

### 3.2. Western Blot in Spinal Cord Tissue

**Figure 2:**
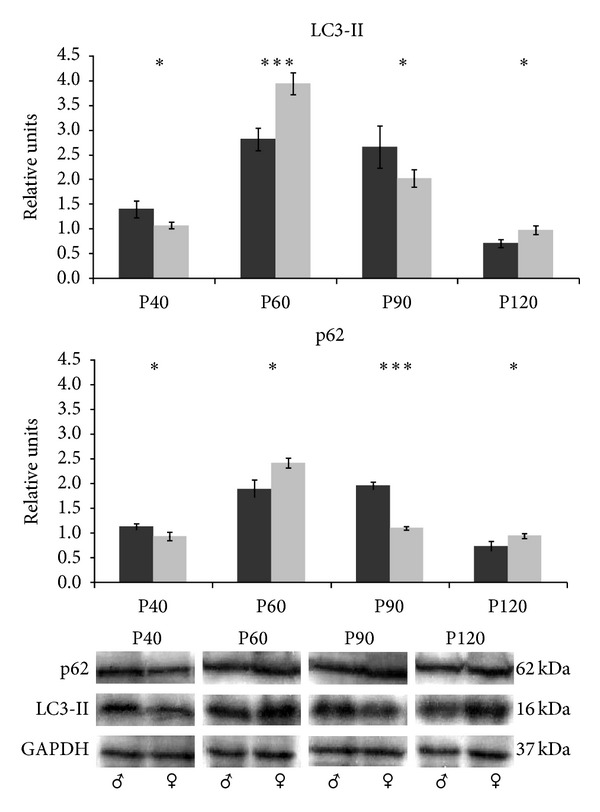
LC3-II and p62 protein expression in the spinal cord. Western blot for LC3-II and p62 proteins in spinal cord tissue from male (black bars) and female (grey bars) mice and its quantitative analysis relative to GAPDH. The representative blots for each age, sex, and protein are shown at the bottom. Data show mean ± SEM of twelve mice. *p* value <0.05 (*) and <0.001 (***).

The immunoblot analysis of spinal cord extracts revealed that LC3-II and p62 protein levels in females increased until P60 and later decreased gradually until P120 (Figure 2). In males, the LC3-II highest expression levels were reached at P60 and the most significant differences between males and females were detected at this age (***p < 0.001). At P40 and P90, LC3-II expression levels were significantly lower in females (*p < 0.05). Moreover, at P60, LC3-II, and p62 protein levels in females reached the maximum levels and significant differences between males and females were detected (***p < 0.001 and *p < 0.05, resp.). The males showed the highest expression levels of p62 at P90 and, in case of p62 protein, the males even doubled the expression levels of females (***p < 0.001).

### 3.3. Western Blot in Skeletal Muscle Tissue

**Figure 3:**
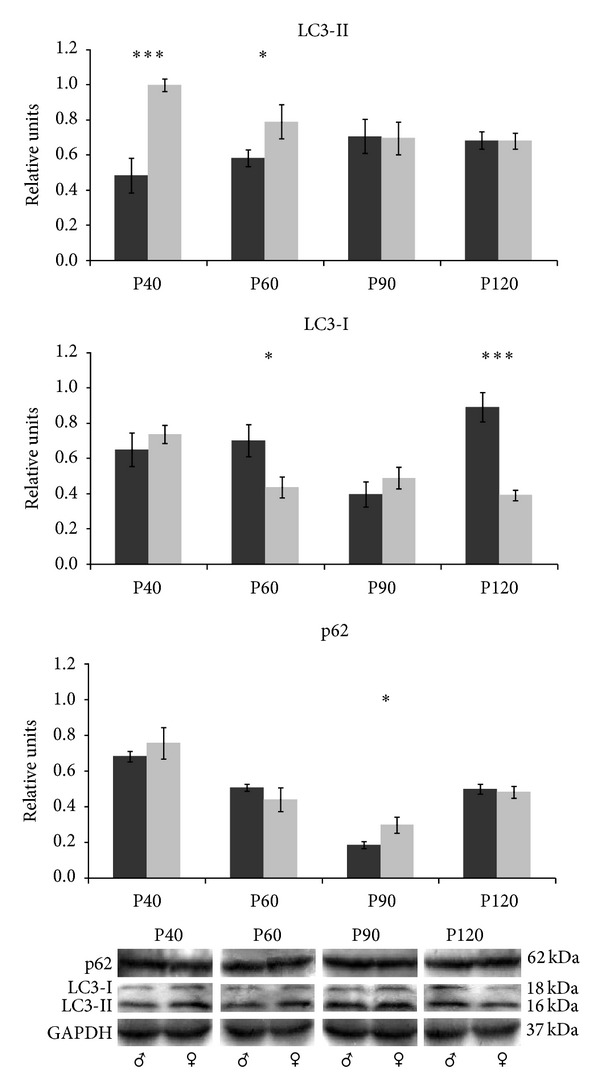
LC3-II and p62 protein expression in the skeletal muscle. Western blot for LC3-II, LC3-I, and p62 proteins in muscle tissue from male (black bars) and female (grey bars) mice and its quantitative analysis relative to GAPDH. Data show mean ± SEM of twelve mice. *p* value <0.05 (*) and <0.001 (***).

Interestingly, LC3 western blot analysis of skeletal muscle tissue revealed two bands corresponding to LC3-I and the activated form, LC3-II (18 and 16 kDa, resp.) (Figure 3). The expression of LC3-II in males increased until P90, although it was similar along the studied ages. In females, the highest LC3-II expression was at P40 and significant differences between males and females were detected at P40 and P60 (***p < 0.001 and *p < 0.05, resp.). LC3-I was not detectable in all the replicate samples and its expression was quite variable. At P60 and P120, females showed lower LC3-I levels than males (*p < 0.05 and ***p < 0.001, resp.). The expression of p62 was not affected by sex at P40, P60, and P120. However, at P90, females showed higher expression levels than males (*p < 0.05).

## 4. Discussion

Experimental studies have demonstrated sex-dependent physiological, morphological, and hormonal differences, and therefore the capability of biological response can be different in both sexes [14]. A number of studies have confirmed sexual dimorphism under pathological and various physiological conditions, underlining the need to include both sexes in the experimental groups. The inclusion of female sex in basic research and clinical trials, such as drug trials, has been essential to reveal potentially different responses and minimize the possibility of adverse sex-dependent effects [15].

This issue was particularly relevant in the case of diseases, in which gender had already been shown to modify the associated pathology, the age of onset, disease progression, or the efficiency of the treatment [16]. Many diseases have been associated with alterations in autophagy and consequently some gender-associated differences have already been demonstrated [16]. For instance, in neurodegenerative disorders, estrogen was neuroprotective and enhanced neurotrophic/synaptic plasticity [17]. In vascular biology, compelling data indicated that sex differences were not only determined by sex steroid levels but may also be modified by innate cellular differences between males and females [18].

Regarding autophagy, only few studies allow for the possible influence of sex-dependence in this molecular process. One of the studies proposed the autophagy as a survival mechanism in males, whereas in female's autophagy resulted in detachment-derived cell death [11]. In accordance with these studies, it has been reported recently that sex differences in constitutive autophagy was found in rats and, moreover, this sexual dimorphism was organ specific [9]. Furthermore, *in vitro* studies also showed a sexual dimorphism in autophagic processes in cancer [12, 13] and in neurons under starvation [4].

Taking into consideration these reported findings, in the present study, differences in constitutive autophagy between males and females of wild type mice are shown. Since the selected ages of the animals in this study have been accurately studied to follow their maturation progress in physiological conditions, in which no influence of disease takes place, the different features displayed by both sexes in this study could be mainly due to hormonal regulators, as previously suggested by Maselli et al. [19]. Significant differences of LC-3 and p62 transcriptional expression levels in spinal cord and skeletal muscle tissues during sexual maturation of the animals have been observed. Furthermore, these differences appeared also at protein levels, in particular at P60 and P90 in spinal cord, in which sexual maturation was completed and at the early stages of maturation process in skeletal muscle. Moreover a different pattern of expression in spinal cord and muscle and, only in the case of muscle tissue, LC3-I protein levels (inactive form of LC3) were detected. Accordingly with previous studies [9], this result suggested a tissue-dependent modulation mechanism of autophagy under physiological conditions.

## 5. Conclusions

We conclude that sex and tissue specific differences exist in physiological autophagy from juvenile to young mice. Therefore, a better knowledge of the wild type mice profile pattern is needed to guarantee a correct interpretation of the results. These results have special importance as they suggest that the sex must be taken into account in the experimental design in order to minimize potential adverse consequences of sex bias.
